# Novel Whole-Cell Inactivated *Neisseria Gonorrhoeae* Microparticles as Vaccine Formulation in Microneedle-Based Transdermal Immunization

**DOI:** 10.3390/vaccines6030060

**Published:** 2018-09-04

**Authors:** Rikhav P. Gala, Rokon Uz Zaman, Martin J. D’Souza, Susu M. Zughaier

**Affiliations:** 1 Vaccine Nanotechnology Laboratory, Department of Pharmaceutical Sciences, College of Pharmacy, Mercer University, Atlanta, GA 30341, USA; rikhav.praful.gala@gmail.com (R.P.G.); rokonuz.zaman@live.mercer.edu (R.U.Z.); DSOUZA_MJ@mercer.edu (M.J.D.); 2 Department of Basic Medical Sciences, College of Medicine, Qatar University, Doha 2713, Qatar

**Keywords:** *Neisseria gonorrhoeae*, gonorrhea, vaccine, microneedle, skin patch, nanotechnology, antigen-specific antibody, antigen-specific CD4 T lymphocytes

## Abstract

*Neisseria gonorrhoeae* is a strict human pathogen responsible for more than 100 million new sexually transmitted infections worldwide each year. Due to the global emergence of antibiotic resistance, the Center for Disease control (CDC) recently listed *N. gonorrhoeae* as an urgent threat to public health. No vaccine is available in spite of the huge disease burden and the possibility of untreatable gonorrhea. The aim of this study is to investigate the immunogenicity of a novel whole-cell-based inactivated gonococcal microparticle vaccine formulation loaded in dissolvable microneedles for transdermal administration. The nanotechnology-based vaccine formulation consists of inactivated whole-cell gonococci strain CDC-F62, spray dried and encapsulated into biodegradable cross-linked albumin matrix with sustained slow antigen release. The dry vaccine nanoparticles were then loaded in a dissolvable microneedle skin patch for transdermal delivery. The efficacy of the whole-cell microparticles vaccine formulation loaded in microneedles was assessed in vitro using dendritic cells and macrophages as well as in vivo mouse model. Antibody titers were measured using an enzyme immunosorbent assay (ELISA) and antigen-specific T lymphocytes were assessed in spleens and lymph nodes. Here we report that whole-cell-based gonococcal microparticle vaccine loaded in dissolvable microneedles for transdermal administration induced significant increase in antigen-specific IgG antibody titers and antigen-specific CD4 and CD8 T lymphocytes in mice compared to gonococcal antigens in solution or empty microneedles. Significant increase in antigen-specific IgG antibody levels was observed at the end of week 2 in groups that received the vaccine compared to the group receiving empty nanoparticles. The advantages of using formalin-fixed whole-cell gonococci that all immunogenic epitopes are covered and preserved from degradation. The spherical shaped micro and nanoparticles are biological mimics of gonococci, therefore present to the immune system as invaders but without the ability to suppress adaptive immunity. In conclusion, the transdermal delivery of microparticles vaccine via a microneedle patch was shown to be an effective system for vaccine delivery. The novel gonorrhea nanovaccine is cheap to produce in a stable dry powder and can be delivered in microneedle skin patch obviating the need for needle use or the cold chain.

## 1. Introduction

*Neisseria gonorrhoeae* is strictly a human pathogen that causes sexually transmitted infection. The disease state termed gonorrhea accounts for >100 million cases worldwide each year. The gonococcus (GC) is noted for its capacity to develop resistance to antibiotics used in therapy [1,2]. The gonococcus can survive extracellularly and intracellularly, however, in both environments, the bacteria must adapt to pressures exerted by the host [3,4]. There were over 400,000 reported cases in the US in 2015, and several more that are not reported [1,2,5]. The disease state termed gonorrhea accounts for >100 million cases worldwide each year. There were over 460,000 reported cases in the US in 2016, and several more that are not reported. It is much more common in Africa and other developing nations [6]. Untreated gonococcal infection in women may progress to pelvic inflammatory disease, increasing the risk of ectopic pregnancy and infertility [7]. Currently, there are no vaccines for gonorrhoeae. The main reason to warrant the development of a gonococcal vaccine is the emergence of antibiotic-resistant GC, which has led to a rapid increase in the prevalence of the infections since 2012 [8]. With the development of antibiotic-resistant strains of *N. gonorrhoeae*, the Food and Drugs Administration (FDA) and CDC have listed the research and development of a vaccine against gonorrhea as a high priority.

To date, no FDA-approved vaccine against gonorrhea is available in spite of the huge burden of disease [9,10,11]. Only two vaccines for *N. gonorrhoeae* have entered into clinical trials in the past. The first was a crude, killed whole-cell vaccine, which was studied in a controlled experiment in a population of Inuit in northern Canada with high incidence and prevalence of *N. gonorrhoeae* infection [12,13]. There was no evidence for protection, even though the vaccine was said to be well tolerated. Although the vaccine induced an antibody response in over 90% of vaccine recipients it lacked the generation of an adaptive immune response which led to the failure of the vaccine study [14]. *N. gonorrhoeae* can interact with various immune cells to elicit innate inflammatory responses and suppress T helper cell Th1/Th2-mediated specific immune responses [15]. Phagocytosis by macrophages results in the activation of NLRP3 inflammasomes, the production of IL-1β, activation of polymorphonuclear neutrophils (PMNs), and activation of cathepsin B, which leads to pyronecrosis of antigen presenting cells (APCs) [16]. Interactions with dendritic cells (DCs) lead to up-regulation of PDL-1 and PDL-2, which induce apoptosis of cells bearing PD-1. This up-regulation also causes the release of IL-10, which has immunoregulatory properties and stimulates type-1 regulatory T cells (Treg1) [15]. Interaction with CD4^+^ T helper cells induces secretion of IL-10, TGF-β, and IL-6 [17]. Activation of Treg1 cells by IL-10 and TGF-β leads to suppression of Th1 and Th2 cells. TGF-β and IL-6 drive the development of Th17 cells, which secrete IL-17 and IL-22, leading to the recruitment or induction of innate defenses such as PMNs and anti-microbial peptides [18]. *N. gonorrhoeae* evades the immune system by PMNs and anti-microbial peptides while concomitantly suppressing the development of adaptive immune responses such as *N. gonorrhoeae* -specific antibodies that could enhance phagocytosis and intracellular clearance of gonococci by phagocytes and bacteriolysis through the classical complement pathway [4,9,19]. 

We developed a novel nanotechnology-based vaccine formulation consisting of formalin fixed whole-cell *N. gonorrhoeae* as the vaccine antigen encapsulated in biodegradable microparticles loaded in microneedles for transdermal administration. The nanotechnology-based vaccine formulations enhance immune responses by slowly releasing antigens and thereby, act as antigen depot. The advantages of using formalin fixed whole-cell gonococci that all immunogenic epitopes are covered and preserved from degradation; the spherical shaped micro and nanoparticles are biological mimics of gonococci, therefore present to the immune system as invaders but without the ability to suppress adaptive immunity; and less toxicity due to lower antigen loading (10%) in nanoparticles. Here we report that the gonococcal nanovaccine formulation enhanced antibody response and induced antigen-specific CD4 and CD8 T lymphocyte in mice vaccinated with gonococcal nanoparticles loaded in microneedles compared to mice vaccinated subcutaneously with gonococcal antigens in solution or empty microneedles.

## 2. Materials and Methods

### 2.1. Preparation of the N. gonorrhoeae Vaccine Antigen

*N. gonorrhoeae* strain CDC-F62 was grown from freezer stock on Gonococcal Base (GCB) agar containing defined Supplement I and II under 5.0% CO_2_ at 37 °C as described [3,4]. Palliated colonies were selected and further subcultured on a GCB agar plate overnight. The fresh growth of piliated colonies was used to inoculate two flasks each containing 300 mL of GCB broth containing defined Supplement I and II with sodium bicarbonate (0.043%) in a 1000 mL sterile flask. The starting OD600~0.2 and the flasks were incubated in a water bath with shaking at 37 °C till late mid-log growth phase i.e., when OD600 reached~0.5. The growth of gonococci was stopped by the addition of 10% formalin (*v*/*v*) and left to mix with gentle shaking overnight at room temperature. The formalin-fixed gonococcal pellets were harvested by centrifugation at 5000× *g* for 15 min at 4 °C. The harvested pellets were washed three times with 45 mL of sterile PBS and centrifuged as above. The final collected pellets were pooled and vortexed thoroughly and saved as a very dense suspension at −80 °C till further use to formulate into vaccines. The particulates were prepared as previously described method established by our laboratory using the Buchi Mini Spray Dryer B-290 [20,21]. These biodegradable particles were characterized for their surface morphology, size, charge, and yield.

### 2.2. Preparation of the N. gonorrhoeae Vaccine Loaded Microparticles

The biodegradable microparticles were prepared following a method previously developed in our vaccine nanotechnology laboratory at Mercer University using the Buchi Mini Spray Dryer B-191 [20,21,22,23,24,25,26,27,28]. Briefly, for a batch of 100 mg of vaccine microparticles at 1% antigen loading, 10 mg of the formalin-fixed whole-cell of *N. gonorrhoeae* (5 mL of 2 mg/mL stock solution) and 90 mg of pre-cross-linked bovine serum albumin (BSA) were mixed. Pre-cross-linked BSA solution was prepared by dissolving 90 mg BSA in 5 mL DI water in a 50 mL beaker. Once BSA is dissolved, glutaraldehyde (25% in DI water purchased from Fisher Scientific, Pittsburgh, PA, USA) was added at 200 µL for every 1 gm of BSA and kept stirring at 300 rpm overnight in a dark place at room temperature. Excess glutaraldehyde was neutralized with sodium bisulphate (10 mg). At this point, the formalin-fixed whole-cell of *N. gonorrhoeae* was added to the pre-cross-linked BSA prepared overnight. 100 mg of this prepared formulation was dissolved in 10 mL of DI water. This formulation was spray dried through a 0.5 mm nozzle (nozzle temperature: −5 °C). The inlet temperature was 120 °C with the aspirator at 100% and a flow rate of 20 mL/h to obtain the *N. gonorrhoeae* vaccine microparticles. 

### 2.3. Microparticle Recovery Yield

Recovery yield of the microparticles after spray drying was calculated for all the batches formulated [27,28,29]. Percent recovery yield was evaluated using the following formula (1):

(1) $$\mathrm{Percentage}\;\mathrm{Recovery}\;\mathrm{Yield}\;\left(\%\right)\;=\frac{\mathrm{The}\;\mathrm{weight}\;\mathrm{of}\;\mathrm{microparticles}\;\mathrm{after}\;\mathrm{spray}\;\mathrm{drying}\times 100}{\mathrm{The}\;\mathrm{weight}\;\mathrm{of}\;\mathrm{all}\;\mathrm{ingredients}\;\mathrm{before}\;\mathrm{spray}\;\mathrm{drying}}$$

### 2.4. Particle Size Distribution

The particle size of the optimized formulation was evaluated using the Spectrex Laser Particle Counter that works on the principle of laser diffraction as previously described [21,30]. Briefly, two mg of the particles were suspended in 1 mL of deionized water, vortexed well, vortexed well for 2 min, and then analyzed by laser diffraction on the particle counter. Particle size was measured in triplicate for empty as well as antigen-loaded particles and contrasted.

### 2.5. Zeta Potential Measurement

Five micrograms of microparticles were suspended in 1 mL of deionized water, transferred to a zeta potential measurement cuvette, and measured using a Malvern Zetasizer. Zeta potential was measured in triplicate for the control formulation and contrasted with the antigen-loaded microparticles [31].

### 2.6. Scanning Electron Microscopy of the Microparticles

Scanning electron microscopy (SEM) was performed to evaluate microparticle size distribution and surface morphology. Microparticles were mounted onto metal stubs using double-sided adhesive tape. After being vacuum-coated with a thin layer (100–150 Å) of gold, the microparticles were examined by a scanning electron microscope Phenome benchtop SEM, Nanoscience Instruments (Phoenix, AZ, USA).

### 2.7. Nitric Oxide Release from Dendritic Cells DC2.4 Cell Line

Dendritic cells (DC2.4) were a kind gift from Dr. Kenneth L. Rock (Dana–Farber Cancer Institute, Inc., Boston, MA, USA). Freshly grown adherent DC2.4 cells were harvested, washed and re-suspended in Dulbecco’s complete media, counted and adjusted to 10^6^ cell/mL 250 µL aliquots were then dispensed into each well of a 48-well plate at final 2.5 × 10^5^ cell density prior to stimulation with gonorrhea vaccine microparticles and blank microparticles. The induced dendritic cells were incubated overnight at 37 °C with 5% CO_2_ and supernatants were harvested. Nitric oxide release was quantified using the Greiss chemical method as previously described [32]. Briefly, the Griess chemical method was used to detect nitrite (NO_2_) accumulated in supernatants of induced RAW264 macrophages. Griess reagent was freshly prepared by mixing equal volumes of 1% sulfanilamide and 0.1% *N*-(1-naphthylethylenediamine) solutions. One hundred microliters of cell supernatants were transferred into a 96-well plate to which 100 µL of Griess reagent was added. The plate was mixed gently, incubated for 10 min at room temperature, and read at 540 nm using a microplate reader (EL312e; BIO-TEK Instruments, Winooski, VT, USA). The optical densities were correlated to the concentration of nitrite. Nitrite was quantitated using the standard curve of sodium nitrite (1 mM stock concentration in distilled water further diluted to the highest standard at 100 µM followed by serial dilutions to 1.56 µM) [32].

### 2.8. Cytotoxicity Study

The toxicity of the vaccine microparticles toward murine RAW264 macrophages (RAW264.7-TIB-71^TM^ cell line was obtained from ATCC, Manassas, VA, USA) was examined in three replicates by the Alamar Blue assay [20,21]. Briefly, 2.5 × 10^3^ cells were plated in each well of a 48 well plate and vaccine microparticles ranging in concentration from 50 µg to 500 µg, with 4 replicates for each concentration, were added to each well. Atropine sulfate was used as a positive control at a concentration of 10 mg/mL. The readings were normalized with the blank microparticles. After 24 h, 10 µL of a 10× solution of Alamar Blue was added to each well and plates were incubated for 4 h at 37 °C following which the fluorescence was measured at 585 nm using Bio-Tek Synergy H1 plate reader, (Winooski, VT, USA).

### 2.9. Formulation of Dissolvable Microneedles for Transdermal Delivery of Vaccine Particles

Dissolvable microneedles encompass the use of polydimethylsiloxane (PDMS) micromolds, which are made from a master structure of microneedles [33]. Briefly, PDMS solution (Ellesworth adhesives, WI) was poured onto the stainless steel master structure. The particulate vaccine microneedles were made using the following formula in Table 1: Gonorrhea vaccine microparticles herein named GC-MP (10% *w*/*w*—5 mg); Trehalose (25% *w*/*w*); Maltose (25% *w*/*w*); PVA (20% *w*/*w*) and HPMC (20% *w*/*w*). The calculated quantities were used for making 2 microneedle patches. First, PVA, HPMC, Maltose and Trehalose were added to a 1.7 mL microcentrifuge tube containing 150 µL of water and vortexed. The weighed amount of gonorrhoea vaccine particles (5 mg) was then added into the mixture. The molds were then placed in a 50 mL centrifuge tube. Approximately 250 micro liters of the formulation was added to the molds and placed into a 50 mL centrifuge tube. Centrifugation was carried out at a speed of 2000 rpm for 5–10 min. Following centrifugation, 200 µL of a backing layer composed of PVA (20% *w*/*w*), HPMC (20% *w*/*w*), Maltose (25% *w*/*w*) and Trehalose (25% *w*/*w*) in 1 mL of an aqueous mixture was then added into the mold and centrifuged. The molds were then placed in an incubator at 37 °C overnight for drying. The microneedle patch measured 1 × 1 cm in size comprising of 100 microneedles (10 × 10). The microneedle array (10 × 10) was then placed on a 3 × 3 cm adhesive patch much like a “Band-Aid”.

### 2.10. Mice Transdermal Immunization Using Dissolvable Microneedles

The immunogenicity of gonorrhea microparticulate vaccine was evaluated using Swiss Webster (CFW) female mice model. Six- to eight-week-old Swiss Webster (CFW) mice were purchased from Charles River Laboratories, Wilmington, MA, and the animals were acclimatized for one week prior use. Mercer University’s Institutional Committee for the care and Use of Laboratory animals (IACUC) approval # A1504009 carried out the animal experiments as per approved protocols.

For delivering microparticles via the transdermal route, mice skin on the back was shaved two days prior to vaccination. One day prior to vaccination, the remainder of the hair was treated with Nair Hair removal cream (Ewing, NJ, USA) for 10 min and then wiped off with a cotton swab. The vaccine loaded microneedles prepared previously were administered in the skin previously treated. The microneedles patch was applied for 20 min which ensured the delivery of the vaccine transdermally. One prime dose (100 µg) at day 0 and two booster doses (100 µg) were given at week 1 and 2 were administered. The mice were monitored and blood samples were collected at every 2-week interval starting from day 0, week 2, 4, 6, 8 and 10. 

### 2.11. Quantification of Vaccine-Specific Serum Antibody Using ELISA

Blood samples were collected from mice prior to each dose of vaccination. Serum was isolated and analyzed for *N. gonorrhoeae*-specific IgG titers using ELISA method [34]. Briefly, poly-l-lysine coated high binding 96 well plates were coated with formalin fixed whole-cell *N. gonorrhoeae* (vaccine antigen,100 µg/well in coating buffer 200 µL) and kept overnight at 4 °C. The plates were washed with 200 µL/well of washing solution (Tris 50 mM, NaCl 0.14 M, Tween-20 0.05%), then blocked with 4% nonfat dry milk (200 µL) (Biorad, Hercules, CA, USA) for 2 h at 37 °C. After washing, the plates were then incubated with 1: 100 dilution of mice sera. After 2 h of incubation followed by washing, HRP-tagged secondary antimouse goat IgG (AbD Serotec^®^, Raleigh, NC, USA) (100 µL/well) was added to each well, incubated for 1 h and then washed with washing solution again. TMB substrate reagent (3,3′,5,5′-tetramethylbenzidine) (BD OptEIA^TM^, BD Biosciences, CA) (100 µL/well) was added and plates were again incubated for 30 min at 37 °C. The reaction was stopped by addition of 4N H_2_SO_4_ (100 µL/well). The plate was read and the absorbance values quantified at 450 nm using Bio-Tek Synergy H1 microplate reader (Bio-Tek Instruments Inc., Winooski, VT, USA).

### 2.12. Determination of T-Cell and B-Cell-Based Immune Response in Lymphatic Organs

The single cell suspension of the spleens and lymph nodes was made using a 40 µm cell strainer. The viability of the cells was checked using the trypan blue exclusion method by TC10^TM^ automated cell counter (Biorad, Hercules, CA, USA). One mL of viable cells at the concentration of 1 × 10^6^ cells/mL was taken in a 1.7 mL Eppendorf tube. The anti-mouse CD4 PE and anti-mouse CD8a FITC (eBioscience, San Diego, CA, USA) was added to cells at the concentration of 10 µL/mL. The tubes were protected from light and incubated with the marker for 30 ± 5 min over ice. After the incubation, the cells were spun and washed 2 times for 30 s using Hanks–ive buffer (200 µL). Then the cells were resuspended in Hanks + ive buffer (200 µL) and stored on ice in a dark place. Meanwhile, the flow cytometer, BD Accuri™ C6 Plus (BD Accuri Cytometers, Ann Arbor, MI, USA) was started and warmed up. The gate for live cells was set with the stock cells. 5000 events were recorded in the gate for each CD4 and CD8 on the flow cytometer [31]. 

### 2.13. Statistical Analysis

All experiments were performed in quadruplets unless otherwise noted. Mean values ± SD and *p* value (Student’s *t*-test unpaired, two-tail distribution) was determined individually for all experiments with Microsoft Excel software. A *p* value of less than 0.05 was considered to be statistically significant.

## 3. Results

### 3.1. Characterization of Whole-Cell Formalin Fixed N. gonorrhoeae Microparticle Vaccine Loaded in Dissolvable Microneedles

#### 3.1.1. Physical Characterization of *N. gonorrhoeae* Microparticle Vaccine

**Figure 1:**
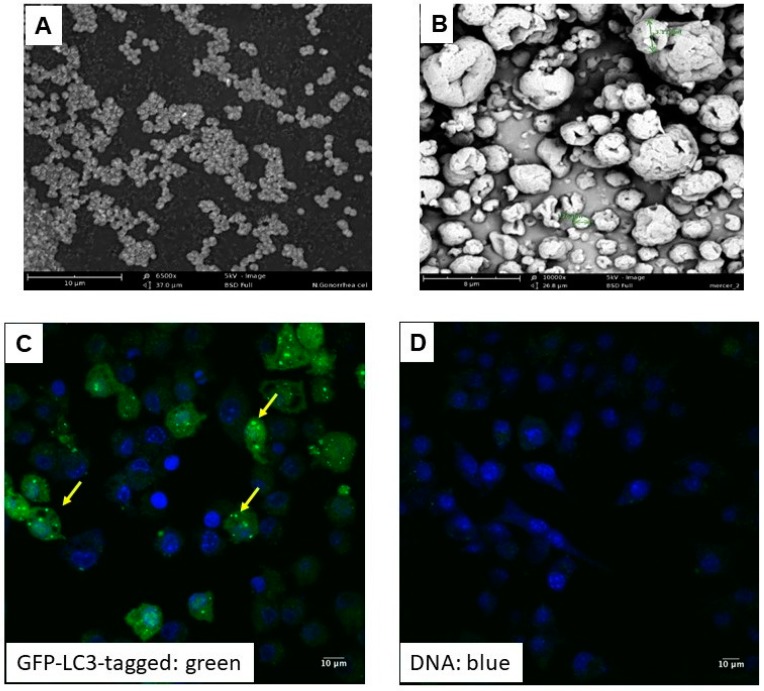
Characterization of formalin fixed whole-cell *N. gonorrhoeae*. (**A**) Scanning electron microscopic (SEM) image of formalin fixed whole-cell *N. gonorrhoeae*, which is the antigen for the vaccine. (**B**) SEM image of spray-dried microparticles containing the *N. gonorrhoeae* vaccine antigen. (**C**) *N. gonorrhoeae* whole-cell vaccine particle uptake by RAW264 macrophages induced autophagic vacuoles visualized using fluorescence microscopy. Green puncta (arrow) are autophagic vacuoles and blue are nucleus stained with DAPI. (**D**) Unstimulated RAW264 macrophages.

The process of fixing whole-cell bacteria using formalin to crosslink bacterial surface structures preserves immunogenic epitopes in their native form, hence not lysed or degraded. In this study, the formalin-fixed whole-cell *N. gonorrhoeae* was intact in native form as examined using the Phenom^®^ Desktop scanning electron microscope under 20 kV at 7500× (Figure 1A). Following spray drying, scanning electron microscopy was also used to observe the particles (Figure 1B). The surface morphology of the formulated microparticles was irregular shaped and rough (Figure 1B). The different shapes of the microparticles may be helpful for uptake by macrophages [35,36,37]. 

The particle size distribution of novel vaccine microparticle formulations from two different batches of empty particles and *N. gonorrhoeae* antigen-loaded microparticles was investigated using Spectrex laser counter (Spectrex Corporation, Redwood City, CA, USA). The average size of the particles ranged from 3.5 µm ± 1.2 µm. There was no significant difference in size between empty and *N. gonorrhoeae* loaded vaccine microparticles ~90% of which were between 1 and 5 µm with an average particle size of 3.65 ± 1.89 µm. The percent yield was found to be 85% after spray drying (*n* = 3 batches, Table 2). The loss during microparticle preparation was due to microparticles sticking to the cylinder and cyclone of the spray dryer. The surface charge was found to be 7.1 mV ± 1.4 mV. Zeta potential is indicative of the surface charge of the particle. A high positive or negative charge indicates good stability and suspendability of the particles when reconstituted in media as it avoids agglomeration [21]. The zeta potential measurements of empty (unloaded) and antigen-loaded microparticle suspensions in deionized water were in the range of −30 to −35 mV with the mean of −32.65 ± 2.4 mV and did not differ significantly from each other (Table 2). Furthermore, The different shapes of the microparticles did not impact uptake by macrophages [35,36,37,38,39]. The uptake of vaccine microparticles by macrophages was assessed using the GFP-LC3-tagged RAW264 cells (a kind gift from Dr. Alfred Merrill, Georgia Institute of Technology, Atlanta, GA, USA), as we previously described [3,21]. Since autophagy plays a crucial role in enhancing antigen presentation, we monitored the ability of ingested microparticles to induce autophagy in macrophages. The uptake of vaccine particles by murine RAW264 macrophages resulted in robust induction of autophagic vacuoles visualized using fluorescence microscopy (Figure 1C) compared to unstimulated macrophages (Figure 1D). The data suggested that the formulated gonococcal vaccine particles are biological mimics of gonococci that retained potential immune stimulatory activity.

**Figure 2:**
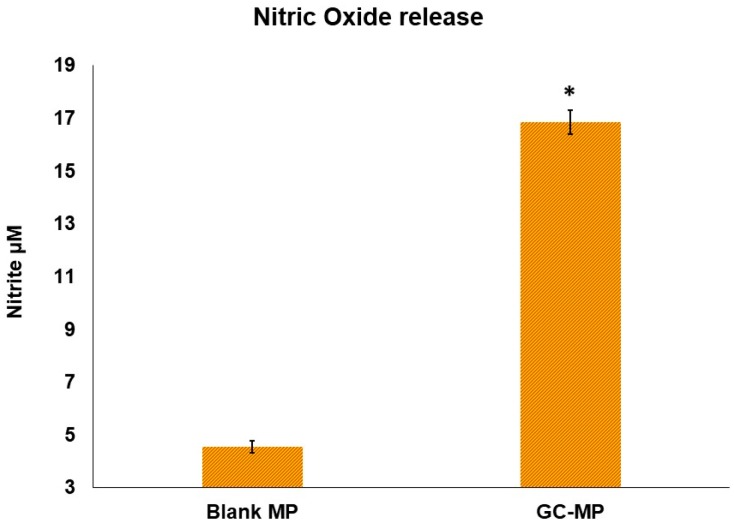
Nitric oxide (NO) production of murine dendritic cells DC 2.4 treated with *N. gonorrhoeae* microparticles. Nitric oxide release from dendritic cells was assessed in murine dendritic cells (DC 2.4) (2.5 × 10^5^) after pulsing with gonorrhea vaccine microparticles for 16 h. Nitrite accumulation in the supernatants was determined using the Greiss reagent. Error bars represent the standard deviation from the average of two independent determinations. The data shown are representative of three independent experiments. Blank MP: empty microparticles; GC-MP: *N. gonorrhoeae*-loaded micropraticles. * *p* ≤ 0.05.

The uptake of vaccine microparticles and the innate immune recognition of vaccine antigens was monitored by assessing nitric oxide release from dendritic cells co-incubated with these particles. Nitric oxide (NO) is an innate immune marker which is released after the uptake and processing of the vaccine antigens reflecting antigen recognition and stimulation of dendritic cells. A higher level of NO release indicates a stronger activation of dendritic cells. We observed a significantly higher level of NO released by the dendritic cells exposed to vaccine microparticles when compared to the blank microparticles (Figure 2). The data suggest that albumin based cross-linked polymer matrix used to make the microparticles are not immunogenic and the innate immune responses generated are attributed to *N. gonorrhoeae* antigen present in the vaccine-loaded microparticles. 

#### 3.1.2. Cytotoxicity Study

**Figure 3:**
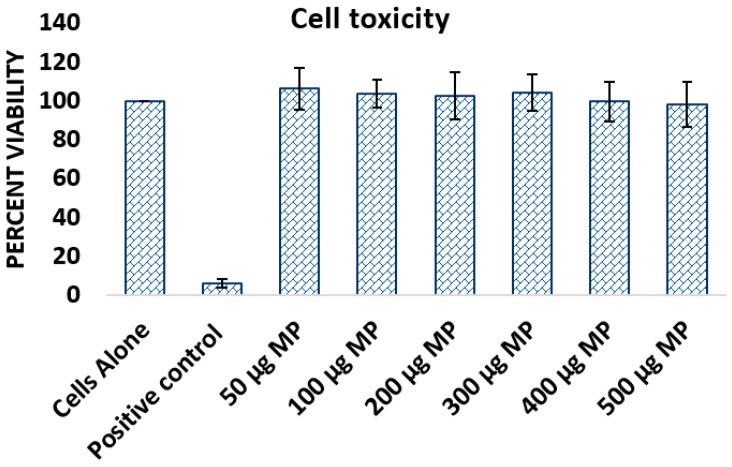
Dosage dependent toxicity test of microparticles on RAW264 murine macrophages cell line. Albumin based microparticles are not toxic to macrophages. RAW264 murine macrophages treated with increasing doses of microparticles and incubated overnight. RAW264 murine macrophages treated with increasing doses of microparticles and incubated overnight. The cytotoxicity was analyzed by the Alamar Blue assay that uses the reducing power of living cells to quantitatively measure cell viability. Atropine sulphate is the positive control and cell only serves as the negative control. Vaccine-loaded microparticles were not cytotoxic for the studied concentrations ranging between 50 to 500 µg. Results are expressed as mean ± standard error (*n* = 3).

To assess the cytotoxicity of the formulated vaccine-loaded microparticles on antigen presenting cells, we employed the Alamar Blue assay [20,21]. The results of the cytotoxicity study indicated that the formulation of gonorrhea vaccine loaded microparticles was not toxic to murine macrophages RAW264 at doses ranging from 50 to 500 µg per well (Figure 3). The viability of the cell population exposed to different doses of microparticles did not differ significantly from the cell populations not exposed to microparticles indicating that the microparticles were not toxic to the cells (Figure 3). Atropine sulfate was used as a positive control and as expected revealed a highly decreased viability in comparison to the negative control, i.e., cells alone. The results indicate that gonorrhea vaccine-loaded microparticles are not toxic to macrophages.

#### 3.1.3. Characterization of the Dissolvable Microneedles

**Figure 4:**
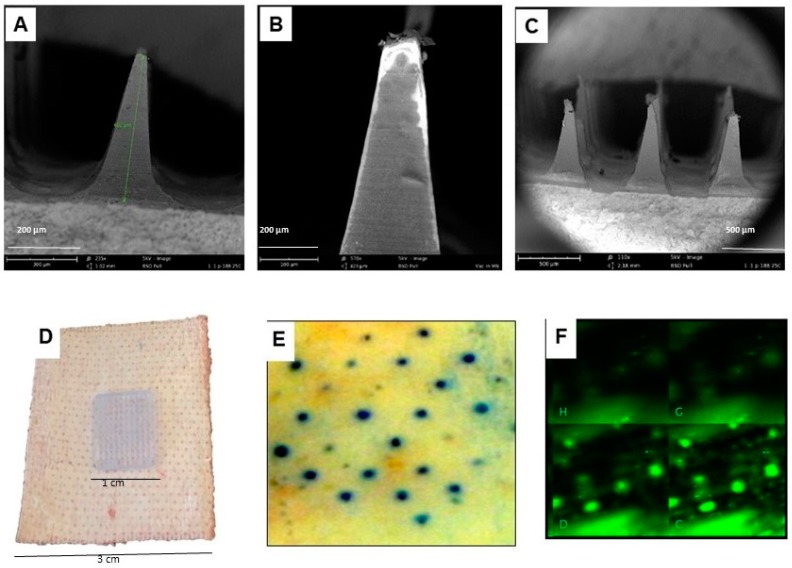
Scanning electron microscopy (SEM) image of the *N. gonorrhoeae* vaccine microparticles loaded in dissolvable microneedles. (**A**) Formulated microneedle is 600 µm in length. (**B**) *N. gonorrhoeae* microparticles loaded in microneedle. (**C**) dissolvable microneedles after transdermal delivery. (**D**) Microneedle skin patch “Band-Aid”. (**E**) Microchannels created by the microneedles patch on the skin using methylene blue dye. (**F**) Z-stack of calcein-stained microchannels as seen on mouse skin using confocal microscopy. Calcein was observed in microchannels to up to 600 ± 60 µm depth.

Scanning electron microscopy was carried out to observe the surface morphology and formation of microneedles. Figure 4A–C shows scanning electron microscopy images of the formulated dissolvable microneedle. The microneedles were pyramid shaped and 600 µm in height (Figure 4A). The fabrication of microneedles was designed to have the vaccine microparticles in the needless of the microneedles patch, allowing it to dissolve in the skin post-application (Figure 4B). This would ensure the vaccine microparticles are being accurately delivered. This microneedle patch consisted of 100 (10 × 10) micron-sized needles in 1 square centimeter (Figure 4C). For transdermal vaccination using microneedle skin patch (Figure 4D), it was important to visualize the microchannels in-order to understand their depth within the skin layers. In order to visualize the microchannels, they were stained with either methylene blue or calcein dye (FluoSpheres^®^ 0.2 μm) and observed by light and confocal microscope respectively (Figure 4E,D). For methylene blue staining, full thickness murine skin was freshly excised from the animal and treated with microneedle patch. The microchannels were stained with 1% *w*/*v* methylene blue solution for 1 min. Excess stain was wiped with a Kimwipe followed by an alcohol wipe. Stained microchannels were imaged with Canon digital camera. A control was also maintained without microneedle treatment to ensure that untreated skin restores the anatomical structure. Both treated and untreated skin samples were embedded in OCT medium in embedding molds and frozen at −80 °C. The frozen skin section was cryomicrotomed transversally using Microm HM505E cryostat (Thermo Scientific, Waltham, MA, USA) with a thickness of 50 μm. These sections were mounted onto glass slides and viewed under the Leica DM750 light microscope using a Leica ICC50HD camera at 10× and 40× magnification (Figure 4E). For confocal microscopy, full thickness skin was freshly excised and treated with microneedles as described previously [40,41]. The channels were then stained with FluSpheres^®^ 0.2 μm for 2 min. Full-thickness skin sections were mounted on microscope slides and viewed under a Zeiss LSM510 confocal microscope with 10× air objective. An argon laser of 488 nm wavelength was used to excite the fluorophore and a band pass filter of 500–550 nm was used (Figure 4F). Image J software (https://imagej.nih.gov) from National Institute of Health, USA was used to analyze the images. 

### 3.2. Immunogenicity of Whole-Cell N. gonorrhoeae Nanovaccine Administered via Subcutaneous Immunization in Mice

**Figure 5:**
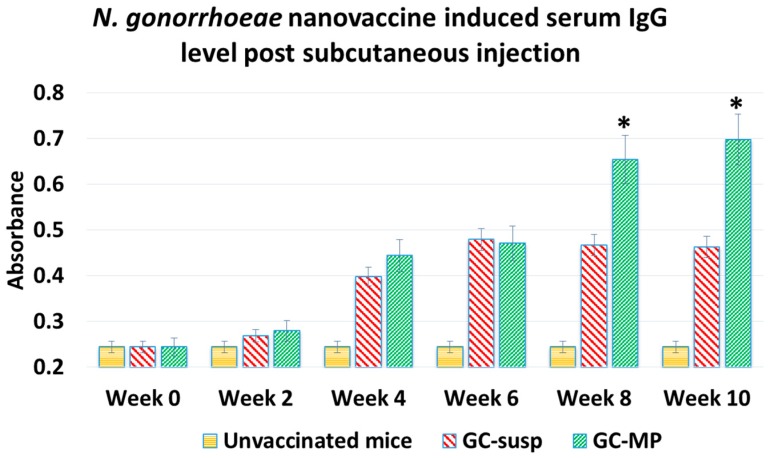
*N. gonorrhoeae*-specific antibody measurement in serum using ELISA. The mice received one prime dose subcutaneously at week 0 followed by 2 booster doses at week 1 and 2. The vaccinated mice groups receiving either GC suspension or GC microparticles vaccines showed significantly higher serum IgG levels from week 4 till week 10, when compared to the unvaccinated mice group (*p* < 0.05). At week 8 and 10, the group receiving microparticulate vaccine showed significantly higher response compared to the subcutaneous vaccine group (* *p* < 0.05). GC-susp: *N. gonorrhoeae* antigen in suspension; GC-MP: *N. gonorrhoeae* antigen-loaded in microparticles.

In order to assess the efficacy of the whole-cell particulate vaccine, 10 mg of particles were weighed containing 500 µg of the antigen and administered to 6–8 week-old Swiss Webster (CFW) subcutaneously. There were three groups in the study, one group receiving the subcutaneous gonorrhea particulate vaccine (GC-MP SubQ), one group receiving subcutaneous gonorrhea vaccine in suspension (GC-susp—500 µg), and a negative control group, which received the blank particles (*n* = 6). The study dosing included one prime dose at week zero, followed by two booster doses at weeks 4 and 6. Blood samples were collected prior to prime dose and every two weeks after dosing. The antibody levels in the blood were measured using specific indirect ELISA [39,40,41]. A rise in gonococci-specific antibody levels was observed beginning at week 4 in groups that received the vaccine compared to the group receiving blank particles (Figure 5). 

### 3.3. Mice Immunization Study Using Microneedles for Transdermal Vaccine Delivery

**Figure 6:**
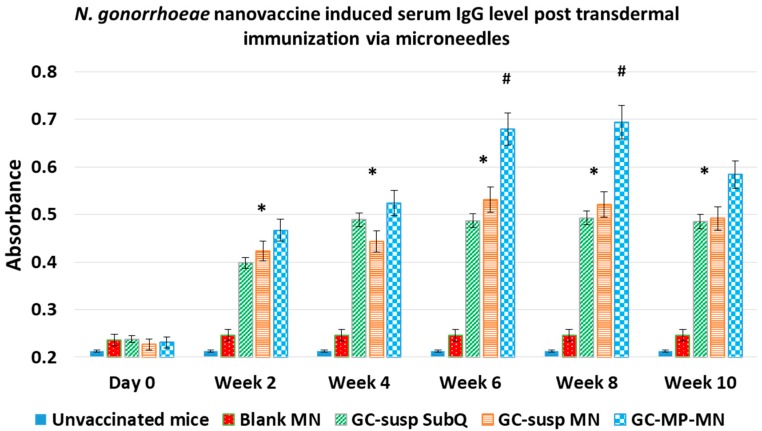
*N. gonorrhoeae*-specific antibody measurement in mice serum immunized transdermally using microneedles patch. Mice were vaccinated at day 0 followed by two booster at week 1 and 2 and immune response was monitored for 10 weeks. ELISA method was used to measure induced serum IgG levels in vaccinated mice and control groups. The groups receiving vaccine showed significantly higher serum IgG titers when compared to the controls i.e., blank microparticles and blank microneedles (MN) after week 2. The group which received the gonorrhea vaccine microparticles in microneedles (GC-MP-MN) showed significantly higher antibody titers than the other 2 vaccine groups at week 6 and 8 (*n* = 6) (* *p* < 0.001; # *p* < 0.05). GC-susp SubQ: *N. gonorrhoeae* antigen in suspension administered subcutaneously (GC-susp SubQ); GC-susp MN: *N. gonorrhoeae* antigen in suspension administered transdermal via microneedle; GC-MP-MN: *N. gonorrhoeae* antigen loaded in nanoparticles administered transdermal via microneedles.

Based on the positive results from the pilot study of mice vaccination via the subcutaneous route, the immunogenicity of the formulated gonorrhea nanovaccine in dissolvable microneedle for transdermal delivery was further investigated. Using the immune system of the skin, we delivered the vaccine particles via a microneedle patch. These microneedles were loaded with gonorrhea microparticles vaccine, which was applied into the skin, much like a “band-aid patch” delivering the vaccine into the skin. The study was carried out in 6–8 week in CFW mice. The following groups were used in the study: (1) Unvaccinated mice; (2) Blank Microneedles (MN); (3) Vaccine Suspension administered via subcutaneous route (GC-susp SubQ); (4) Vaccine suspension loaded Microneedles administered via transdermal route (GC-susp MN); (5) Vaccine microparticles in Microneedles administered via transdermal route (GC-MP-MN). One prime dose (week zero) and two booster doses (week 2 and 4) were administered, and blood samples were collected prior to dosing and every 2 weeks after dosing. The animals were monitored for 10 weeks. The antibody was measured as previously described using an ELISA method, which demonstrates significantly higher serum IgG titers in groups receiving the gonorrhea (GC) vaccine when compared to the controls (blank particles and blank microneedles after week 2) (Figure 6). The group that received the GC vaccine microparticles in microneedles showed significantly higher antibody titers at weeks 6 and 8 compared to other groups, which received the GC vaccine in suspension administered subcutaneously (GC-susp SubQ). Our data demonstrate that the transdermal delivery of a microparticles GC vaccine loaded in the microneedle (GC-MP-MN) patch was efficient and effective for vaccine delivery. 

### 3.4. Induction of Antigen-Specific CD4 and CD8 T Lymphocytes

**Figure 7:**
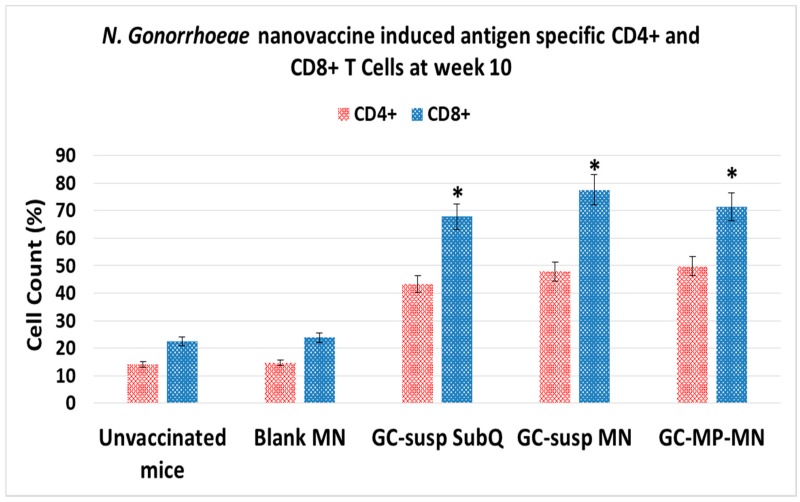
*N. gonorrhoeae* microparticle vaccine induced antigen-specific CD4^+^ and CD8^+^ T-cells counts in the splenocytes at week 10 after immunization. Groups receiving the vaccine showed significantly higher CD4^+^ and CD8^+^ T cells than compared to the controls i.e., unvaccinated and blank microneedles (*p* < 0.05). GC-susp SubQ: *N. gonorrhoeae* antigen in suspension administered subcutaneously; GC-susp MN: *N. gonorrhoeae* antigen in suspension administered transdermal via microneedle; GC-MP-MN: *N. gonorrhoeae* antigen loaded in microparticles administered transdermal via microneedles.

Since the gonorrhea microparticle vaccine induced humoral immune responses in mice evidenced by the significant increase in antigen-specific antibody titers, we further investigated whether this vaccine induced antigen-specific T lymphocytes. Briefly, the animals were sacrificed at week 12 and the primary and secondary lymphoid organs were extracted (i.e., spleen and lymph node), and processed into single cell suspensions in order to determine the antigen-specific T lymphocyte responses. The single cell suspensions were stained with fluorescence-conjugated antibodies specific to T cells, helper T cells (CD4^+^) and cytotoxic T cells (CD8^+^) and quantified using flow cytometry. In order to determine the antigen-specific T cell responses, splenocytes from the various groups were plated onto a 48 well plate and re-exposed to the GC antigen (50 µg in 100 µL) for 16 h and then stained with fluorescent tagged antibodies for helper T cells (CD4^+^ PE, eBiosciences, San Diego, CA, USA) and cytotoxic T cells (CD8^+^ FITC, eBiosciences, San Diego, CA, USA) [31]. The cell count percentage was compared with mice treated with blank microneedles. The CD8^+^ and CD4^+^ T-cell populations were found to be elevated in splenocytes of vaccinated mice when compared to naïve and the blank microneedle group (Figure 7). Again, the transdermal delivery of microparticles GC vaccine via a microneedle patch was shown to be an efficient and effective vaccine delivery method. 

## 4. Discussion

We have developed a biodegradable and biocompatible polymer matrix system for making microparticles loaded with vaccine antigens using the spray drying method [20,21,24,41]. We formulated a sustained release particulate gonococcal vaccine that consists of formalin-fixed inactivated whole-cell gonococci encapsulated in an albumin-based polymer matrix that mimics the chemical conjugation process to a protein carrier; hence elicit a T-cell-dependent immune response. The novel particulate gonorrhea vaccine formulation is delivered transdermally using biodegradable microneedles “skin patch”. This novel gonorrhea vaccine skin patch is tested in vivo using mouse model and data demonstrated that transdermal vaccine delivery induced significantly higher levels of humoral and adaptive immune responses i.e., antigen-specific serum IgG and antigen-specific CD4 and CD8 T lymphocytes.

Transdermal vaccine delivery is advantageous and shown to enhance immune responses to vaccine antigens. Skin provides a unique site for the vaccination purposes as it is easily accessible and houses various immune cells for an efficient immune response against a range of antigens. Skin serves as a barrier against various pathogens and is equipped with the skin-associated lymphoid tissues (SALT) to combat any insult from invading pathogens [42]. Various skin cells assist in generation of effective immune response [43]. Keratinocytes are the most predominant (95%) epidermal cells in the skin. The skin harbors a special kind of dendritic cells called the Langerhans cells. Keratinocytes and other cells can be activated by pathogens and result in production of cytokines and chemokines, which in turn recruits dendritic cells or antigen-presenting cells to the site of action leading to initiation of the immune response. Langerhans cells comprise of only 2% of the total cell population in the epidermis but due to their extended dendrites spread in the epidermal layer they cover over 25% of the skin surface. These are professional phagocytic cells efficient in immune surveillance and further signaling to the T-cells present in their vicinity. Activated macrophages and T-cells drain into nearby lymph nodes leading to an enhanced immune response. Currently most of the vaccines are administered via subcutaneous or intramuscular route [33]. These have been highly effective in generating protective immune response but they remain invasive, painful and require a skilled professional for vaccination. In an attempt to minimize some of these issues scientists have explored the potential of delivering vaccine antigens intradermally using microneedles [38,39]. Microneedles, as the name indicates, are micron-sized needles, which upon insertion into the skin result in formation of aqueous conduits forming a passage for the vaccine antigens towards the immune-competent skin layers. Due to their short needle length, they avoid contact with the nerve endings in the dermis thus remain to be a painless mode of immunization. Recently FDA approved Intanza™ (Sanofi Pasteur, Rockville, MD, USA), an intradermal influenza vaccine that incorporates a 1.5 mm needle attached to a pre-filled syringe loaded with flu antigens. It has been shown to be efficacious when compared with an IM flu vaccine thus bringing a switch from hypodermic needles to “micro”-needles for immunizations [44]. This opens a new avenue of vaccine delivery through an effective, painless and patient-friendly route of administration. The success of immunization via skin using microneedles inspired us to evaluate the potential of delivering *N. gonorrhoeae* whole-cell inactivated vaccine via skin patch route. 

Using formalin fixed whole-cell gonococci as vaccine preserved all the possible antigenic proteins in their native form to antigen-presenting cells. This approach will cover all the immunogenic epitopes and help in inducing an immune response. Moreover, when encapsulated in a particulate form, it enhances uptake by the APCs consequently enhancing antigen presentation. Our approach of using particulate-based delivery systems, which is believed to interact distinctively with the immune system by slowly releasing antigens, has shown significant enhancement in immune activation [20,21,41,45]. The particulate nature of the vaccine allows for better antigen uptake by dendritic cells and macrophages, leading to improved antigen presentation and subsequent activation of T cells. The skin is rich in antigen-presenting cells (APCs), known as Langerhans cells (LCs) in the epidermis and dermal dendritic cells in the dermis that can activate T and B lymphocytes, and therefore is an excellent route of delivery for vaccines as shown by previous studies conducted in our laboratory [38,39]. Therefore, the whole-cell *Neisseria gonorrhoeae* in a particulate delivery system delivered via microneedles into the skin will provide an excellent potential immunization strategy against gonorrhea. Thus, optimizing a transdermal vaccine formulation that confers protection and provides significant advantages over the conventional antibiotic therapy will have a significant public health impact in the United States.

The role of vaccines in preventing infectious diseases such as pneumococcal and meningococcal diseases, consequently reducing the global burden of disease, has been demonstrated and provided significant public health advantage. The vaccine-based preventive approach proved to be the most cost-effective in reducing disease burden. Therefore, a protective gonococcal vaccine is highly sought to slow the spread of antibiotic-resistant gonococci and to reduce the burden of this STD. However, it is quite challenging to design gonorrhea vaccine that provides 100% protection due to the elusive nature of immune responses elicited during gonococcal infections. In many cases, gonococcal infections are either asymptomatic or silent with mild symptoms, whereas in purulent gonorrhea infections, potent innate immune responses are induced but protective adaptive immune responses are not demonstrated. *N. gonorrhoeae* main surface antigens such as pilin are highly antigenically variable which adds another astounding challenge to developing a protective subunit gonorrhea vaccine [9]. In our study here, we used a whole-cell based gonorrhea as vaccine antigen delivered transdermally which demonstrated the induction of both humoral and adaptive immune responses. The recent observation that meningococcal outer membrane vesicle (OMV) vaccine is cross-protective and lead to a reduction in gonococcal infections adds a new hope to the potential of whole-cell gonorrhea vaccine [46]. Both pathogenic Neisseria species *N. meningitidis* and *N. gonorrhoeae* share conserved immunogenic epitopes in surface structures used in OMV development; therefore, lend support to using whole-cell gonorrhea for vaccine development. When designing a vaccine, it is predicted that an efficacy level of 70% would reduce a considerable amount of the disease burden and transmission worldwide, which will be critical in combating the spread of antibiotic resistance in gonococci [47]. The current comprehensive effort to provide gonorrhea vaccine led by the National Institute of Health (NIH) and World Health Organization (WHO), as well as many public health agencies worldwide, demonstrate the dire need for this vaccine. The continuous global research to understand the mechanisms of host–pathogen interactions would help unravel the mechanism of immune responses against gonococcal infections that help design refined vaccines [48]. 

The use of an inactivated whole-cell based gonorrhea vaccine was attempted in 1974 in studies lead by Greenberg, where two clinical trials were conducted in isolated villages in northern Canada [12,13]. The data showed over 90% of vaccinated subjects developed antigen-specific antibody titers that remained high when measured again after one year. This experimental gonococcus vaccine did not confer 100% protection, as 10% of the vaccinated population experienced repeated gonococcal infections [13]. That particular gonococcus vaccine was prepared in broth culture incubated with thiomersal as a preservative and left to autolyze at room temperature [12,13]. In contrast, our proposed whole-cell gonorrhea vaccine is formalin-fixed, thus preserved from lysis as demonstrated with scanning EM, which suggests that immunogenic epitopes are also preserved from degradation and maintained in their native form. We argue that the use of whole-cell formalin fixed gonococci as a vaccine antigen with preserved epitopes loaded in microparticles and delivered transdermally via microneedles is advantageous over other whole-cell preparations. The dynamics of slow and sustained antigen release using this nanotechnology approach enhances the uptake of antigen and the innate immune responses consequently inducing desired adaptive immune responses. The encouraging data demonstrated that this novel gonorrhea skin patch induced significant adaptive cellular immunity i.e., antigen-specific CD4 and CD8 lymphocytes. Remain to be seen whether this novel whole-cell gonorrhea skin patch vaccine can provide protective immunity upon challenge with isogenic vaccine strain and confer cross-protection against various *N. gonorrhoeae* strains. Ongoing studies are addressing the efficacy of gonorrhea skin patch vaccine in mouse lower genital tract infection model and the bactericidal activity of the induced IgG as well as mucosal IgA levels. Our data presented here is a proof-of-concept that requires fine-tuning and optimization of gonorrhea vaccine antigen qualitatively and quantitatively which is the main limitation of our study. 

However, this study demonstrates the potential of this novel nanotechnology-based vaccine skin patch formulation. We argue that the proposed nanovaccine shelf life is expected to be several folds higher than that of conventional vaccines since it is spray dried and kept well protected from moisture. The novel nanovaccine encapsulated with the whole-cell inactivated *N. gonorrhoeae* incorporated into an albumin-based particulate matrix provides the following advantages: whole-cell based vaccine that encompasses all immunogenic epitopes; self-adjuvanted vaccine formulations enhance immunogenicity with the addition of outer membrane proteins and molecules including endotoxin that are Toll-Like Receptors (TLR) ligands; improved uptake by immune cells and slow antigen release, i.e., antigen depot effect; induction of robust autophagy formation that enhances antigen presentation; heat-stable formulation that does not require refrigeration; administration by a microneedle skin patch which is noninvasive; and reduced cost with the elimination of expenditures relating to identification of a single immunogenic epitope, purification and scale-up of the antigen, as well as individual packaging and refrigeration of ampoules.

## 5. Conclusions

We formulated a novel gonorrhea vaccine consists of biodegradable whole-cell formalin-fixed *N. gonorrhoeae* as an antigen encapsulated in microparticles then loaded in microneedle “skin patch” for transdermal vaccine delivery. The novel gonorrhea nanovaccine activity was characterized by in vitro cell-based studies and an in vivo vaccination pilot study using mice. Our data suggest that we have a potentially functional vaccine that elicited an antigen-specific antibody response and antigen-specific CD4 and CD8 T lymphocyte responses. Further experiments are ongoing to characterize and establish this novel gonorrhea nanovaccine with the addition of adjuvants and determine the correlates of protection in immunized mice.

## Figures and Tables

**Table 1:** Formula for the preparation of microneedles.

Material	Percent *w*/*w*	200 mg Batch
Vaccine microparticles	10%	20 mg
Trehalose	25%	50 mg
Maltose	25%	50 mg
PVA	20%	40 mg
HPMC	20%	40 mg

**Table 2:** Physical characteristics of the gonorrhea vaccine microparticles. The recovery yield (%) after the spray drying process, particle size, and the zeta potential were measured in triplicates and mean and the standard deviation are reported along with the range.

Physical Characteristics	Range	Mean ± SD
Recovery yield (%)	10	91.56 ± 5.3
Particle Size (µm)	4	3.65 ± 1.89
Zeta Potential (mV)	5	−32.65 ± 2.4
